# Intervention, improved prevention, imaging, inflammation, and innovation: the five I's cardiovascular highlights in *EHJ Open* 2024

**DOI:** 10.1093/ehjopen/oeaf015

**Published:** 2025-02-28

**Authors:** Magnus Bäck, Maciej Banach, Frieder Braunschweig, Salvatore De Rosa, Frank A Flachskampf, Thomas Kahan, Daniel F J Ketelhuth, Patrizio Lancellotti, Susanna C Larsson, Linda Mellbin, Gianluigi Savarese, Annette Schophuus Jensen, Karolina Szummer, Denis Wahl, European Heart Journal Open Section Editors, Ana Almeida, Athanase Benetos, Marie-Luce Bochaton-Piallat, Mats Börjesson, Ronny Buechel, Christian de Chillou, Jérémy Fauconnier, Dipender Gill, Nicolas Girerd, Joerg Herrmann, Stefan Koudstaal, Seth Martin, Edit Nagy, Jens Cosedis Nielsen, John Pernow, Davide Stolfo, Giuseppe Vergaro, Faiez Zannad, Michal Zembala

**Affiliations:** Department of Cardiology, Heart and Vascular Center, Karolinska University Hospital, 17176 Stockholm, Sweden; Department of Medicine Solna, Karolinska Institutet, 17176 Stockholm, Sweden; Université de Lorraine, Inserm, DCAC, F-54000 Nancy, France; Centre Hospitalier Régional Universitaire de Nancy, 54505 Vandoeuvre-lès-Nancy, France; Faculty of Medicine, The John Paul II Catholic University of Lublin, Aleje Raclawickie 14, 20-950 Lublin, Poland; Department of Preventive Cardiology and Lipidology, Medical University of Lodz and Polish Mother's Memorial Hospital Research Institute, Rzgowska 281/289, 93-338 Lodz, Poland; Department of Cardiology, Heart and Vascular Center, Karolinska University Hospital, 17176 Stockholm, Sweden; Department of Medicine Huddinge, Karolinska Institutet, 14186 Stockholm, Sweden; Department of Medical and Surgical Sciences, Magna Graecia University of Catanzaro, Viale Europa, snc, 88100 Catanzaro, Italy; Divisions of Clinical Physiology and Cardiology, Uppsala University Clinic, 75185 Uppsala, Sweden; Department of Medical Sciences, Uppsala University, 75185 Uppsala, Sweden; Department of Cardiology, Danderyd Hospital, 18288 Stockholm, Sweden; Department of Clinical Sciences, Danderyd Hospital, Karolinska Institutet, 18288 Stockholm, Sweden; Department of Molecular Medicine, University of Southern Denmark, Campusvej 55, 5230, Odense, Denmark; University of Liège Hospital, GIGA Cardiovascular Sciences, Centre Hospitalier Universitaire Sart Tilman, 4000 Liège, Belgium; Unit of Cardiovascular and Nutritional Epidemiology, Institute of Environmental Medicine, 17177 Karolinska Institutet, Stockholm, Sweden; Medical Epidemiology, Department of Surgical Sciences, Uppsala University, 75185 Uppsala, Sweden; Department of Cardiology, Heart and Vascular Center, Karolinska University Hospital, 17176 Stockholm, Sweden; Department of Medicine Solna, Karolinska Institutet, 17176 Stockholm, Sweden; Department of Clinical Science and Education, Södersjukhuset, Karolinska Institutet, 11883 Stockholm, Sweden; Department of Cardiology, Copenhagen University Hospital - Rigshospitalet, Inge Lehmanns Vej 7, 2100 Copenhagen, Denmark; Department of Cardiology, Heart and Vascular Center, Karolinska University Hospital, 17176 Stockholm, Sweden; Department of Medicine Huddinge, Karolinska Institutet, 14186 Stockholm, Sweden; Université de Lorraine, Inserm, DCAC, F-54000 Nancy, France; Vascular Medicine Department and Reference Center for Rare Systemic Autoimmune and Autoinflammatory Diseases, Centre Hospitalier Régional Universitaire de Nancy, 54505 Vandoeuvre-lès-Nancy, France

*Intervention, Improved prevention, Imaging, Inflammation, and Innovation* are highlights of some major insights from *European Heart Journal Open* 2024. Those I's also put spotlight on five of the future perspectives in cardiology trajectories towards optimization of diagnosis, interventions, and treatments, as well as novel innovative targets and methods.

## Intervention

To balance potent P2Y_12_ inhibitors after percutaneous coronary intervention (PCI), a guided selection of P2Y_12_ inhibiting therapy has been associated with the most favourable balance between efficacy and adverse bleeding effects in a meta-analysis.^1^ In the case of co-morbidities requiring both anti-coagulation and anti-platelet therapy, such as concomitant atrial fibrillation and PCI, a large nation-wide registry study showed a lower ischaemic risk without increased bleeding with ticagrelor or prasugrel over clopidogrel,^2^ which aligns with the real-world PERSEO registry^3^ and supports an individualized choice of P2Y_12_ inhibitors.

Beyond optimizing post-intervention care, novel interventional cardiology technologies also emphasize pre-intervention patient selection to improve treatment success. Mitral valve transcatheter edge-to-edge repair (TEER) significantly reduced the combined endpoint of heart failure hospitalizations and mortality compared with medical therapy in the RESHAPE-HF2 (Transcatheter Valve Repair in Heart Failure with Moderate to Severe Mitral Regurgitation) trial.^4^ The results highlight possible need for a reconsidered severity threshold for secondary mitral regurgitation intervention as well as an integrated approach including also left ventricular remodelling for optimizing patient selection and TEER success.^5^

## Improved prevention

Successful prevention, both before and after an optimized intervention, relies on life-style modifications and pharmacological interventions. Although recent evidence suggests electronic cigarettes as a potential addition to counselling for quitting regular smoking,^6^ a contemporary meta-analysis showed that electronic cigarettes containing nicotine are not without risks and affect the cardiovascular system acutely similar to regular combustible cigarettes.^7^ In addition to life-style changes, polypills may facilitate the attainment of guideline-recommended prevention linked to better outcomes, less side effects, and being cost saving compared with other therapeutic options.^8^ Those results support and extend other findings on the use of polypill in secondary prevention in patients with coronary artery disease.^9^

Lipoprotein(a) [Lp(a)] is emerging in prevention, for risk prediction^10^ and with therapeutic potential.^11^ Although Lp(a) levels are predominantly genetically determined, temporal-related changes in Lp(a) variability suggests that repeat Lp(a) measures allow more precise cardiovascular risk prediction.^12^ Prevention with available lipid-lowering is also challenging in some special populations, such as the need for establishing safety and efficacy of dyslipidaemia treatment during pregnancy.^13^ Finally, improved prevention through lipid lowering also comprises novel targets and potential personalized medicine approaches. A drug-target Mendelian randomization analysis supports lowering plasma ANGPTL3, ANGPTL4, and APOC3 levels as promising novel strategies for reducing cardiovascular disease risk.^14^

## Imaging

Extracellular volume (ECV), a marker of myocardial fibrosis and other conditions, traditionally assessed via cardiac magnetic resonance, can also be evaluated using coronary computed tomography angiography (CTA).^15^ In a study retrospectively evaluating 874 patients who underwent CTA for the evaluation of coronary artery disease myocardial ECV fraction was obtained from the CTA data.^16^ The results detected 12% of patients with an ECV fraction >35%, which was associated with several pathologic conditions, among which cardiac amyloidosis was diagnosed in 15 patients.^16^ This cohort is considerably larger than all studies combined from a recent meta-analysis^15^ and points to ECV fraction as a clinically meaningful parameter extractable from clinically indicated CTA.

## Inflammation

Chronic inflammation is well-established as a residual risk for atherosclerotic cardiovascular disease.^17^ Among the currently implemented anti-inflammatory targets, colchicine inhibits cytoskeletal microtubule formation leading to decreased vesicle transport and secretion, but its use may be limited by adverse effects.^18^ Despite previous outcome trials reporting significantly reduced cardiovascular events with low-dose colchicine^19^ and new ESC recommendations for colchicine in chronic coronary syndromes,^20^ its use remains limited. The most recent results from the CLEAR trial however showed that treatment with colchicine after myocardial infarction for a median of 3 years did not reduce the incidence of the composite primary cardiovascular outcome.^21^ Nevertheless, colchicine decreased CRP compared with placebo,^21^ pointing to decreased inflammation. Colchicine's potential effects on lower-extremity peripheral artery disease (PAD) evaluated in emulation of target trials using a real-world sample of patients with PAD and gout, did not conclude that colchicine decreased the cardiovascular or limb events,^22^ and the ongoing randomized clinical trial LEADER-PAD (NCT04774159) is evaluating the effects of colchicine in PAD.^23^

Among other anti-inflammatory targets, IL-6's broad effects has made it an attractive target for anti-inflammatory cardiovascular risk reduction,^24^ which is currently evaluated in an ongoing trial of the monoclonal anti-IL-6 ziltivekimab (NCT05021835).^19^ Importantly, myeloid-specific IL-6 has been shown to significantly impact vascular function^25^ and to be linked to a newly identified population of monocyte-derived lipid-laden pro-inflammatory macrophages that can accelerate atherosclerosis.^26^ These findings suggest that not only the secreted IL-6 protein but also the cells overexpressing it should be considered for future anti-inflammatory cardiovascular therapies.

## Innovation

Among machine learning methods that enable novel and innovative approaches, spectral regression learning is emerging as a promising technique for advanced data analysis applicable to measures of aortic stiffness, which represents structural changes of the arterial system and associates with future cardiovascular events and mortality.^27^ Wave separation analysis is a tool for decomposing the components of blood pressure waveform, but the need for concurrent measurements of pressure and flow limits its applicability. A recent study introduced a spectral regression learning method for pressure-only wave separation analysis using data from the Framingham Heart Study to evaluate the accuracy of pressure-only estimates, and their association with aortic stiffness, assessed by carotid-femoral pulse wave velocity (cf-PWV).^28^ After adjustments for potential confounders, the results suggested that the pressure-only evaluations of wave separation parameters predicted cf-PWV, with forward wave amplitude being the only significant factor. The authors propose that aortic stiffness estimated through this spectral regression learning and single pressure waveform measurements outperform methods used in previous studies^29^ and provide a valuable non-invasive tool for cardiovascular assessment.

In summary, the 5 I's put forward here highlights *European Heart Journal Open* as an open access forum for providing important insights into the recent development of cardiovascular medicine and surgery. We encourage you to explore the journal's full content, which is freely available, and welcome submissions that contribute to a continued open and interactive cardiovascular forum with global sharing of science.

## Data Availability

There are no new data associated with this article.
